# Please Like Me: Facebook and Public Health Communication

**DOI:** 10.1371/journal.pone.0162765

**Published:** 2016-09-15

**Authors:** James Kite, Bridget C. Foley, Anne C. Grunseit, Becky Freeman

**Affiliations:** Prevention Research Collaboration, Charles Perkins Centre and School of Public Health, University of Sydney, Sydney, Australia; University of Cambridge, UNITED KINGDOM

## Abstract

Facebook, the most widely used social media platform, has been adopted by public health organisations for health promotion and behaviour change campaigns and activities. However, limited information is available on the most effective and efficient use of Facebook for this purpose. This study sought to identify the features of Facebook posts that are associated with higher user engagement on Australian public health organisations’ Facebook pages. We selected 20 eligible pages through a systematic search and coded 360-days of posts for each page. Posts were coded by: post type (e.g., photo, text only etc.), communication technique employed (e.g. testimonial, informative etc.) and use of marketing elements (e.g., branding, use of mascots). A series of negative binomial regressions were used to assess associations between post characteristics and user engagement as measured by the number of likes, shares and comments. Our results showed that video posts attracted the greatest amount of user engagement, although an analysis of a subset of the data suggested this may be a reflection of the Facebook algorithm, which governs what is and is not shown in user newsfeeds and appear to preference videos over other post types. Posts that featured a positive emotional appeal or provided factual information attracted higher levels of user engagement, while conventional marketing elements, such as sponsorships and the use of persons of authority, generally discouraged user engagement, with the exception of posts that included a celebrity or sportsperson. Our results give insight into post content that maximises user engagement and begins to fill the knowledge gap on effective use of Facebook by public health organisations.

## Introduction

In countries with high Internet penetration, many people gather in online communities to share information, knowledge, and opinions; platforms facilitating these gatherings are known collectively as ‘social media’.[1] Social media activities currently include multi-media sharing, service and product review sites, blogging and microblogging, and social networking. Globally, Facebook, a social networking platform, is by far the most widely used social media.[2, 3] For example, in Australia, nearly two-thirds of adults maintain a Facebook profile, compared with less than one-fifth of adults for the next two most popular sites, LinkedIn and Instagram.[2] Additionally, almost 40% of Australian Facebook users log in 20 times or more per week, far exceeding any other platform. Research from the United States has also shown that while the rate of new members joining Facebook may have slowed, engagement has intensified, with over 70% of Facebook users engaging with the site at least once a day and 45% several times a day.[4] Although use of Facebook is greatest among young adults (18–29 years), significant proportions of older adults and adolescents maintain a Facebook profile, underlining the ubiquity of this social media platform. [2, 4]

Facebook is also the most widely used social media platform by businesses.[2] Although an emerging area of research, a recent examination of the most popular Facebook pages of energy dense, nutrient poor food and beverage brands revealed the host companies use a range of marketing techniques, such as interactive games and competitions and prizes based on user-generated content (i.e. content that is created by and posted to the site by users, not administrators), to engage with consumers, with adolescent and young adults the most receptive to these techniques.[5] Another study found similar techniques being used by alcohol brands and noted in particular the strategic use of timing and context to maximise user engagement.[6] Importantly, many of the techniques are unique to social media suggesting a deliberate strategy by these organisations to exploit the bidirectional format of social media and generate maximum interest and engagement with users.

Facebook defines engagement as users reacting to (i.e. ‘liking’), sharing, or commenting or clicking on any content.[7] Generating engagement is important because it not only reflects the ability of the content to capture the attention of users but also directly influences the reach of content.[8–10] Previous research has found that users of social networking sites such as Facebook primarily share information on these platforms when they believe the information is beneficial to others.[11] Researchers argue that this reflects an expectation that the shared information will be beneficial to maintaining and strengthening the users’ online community, thereby creating an incentive to maximise user engagement in order generate word-of-mouth marketing; that is, marketing between consumers.[12, 13]

As Freeman et al. [5] showed, Facebook users who engage with commercial brand pages through liking, sharing, or commenting on content are either unaware or unconcerned that their engagement generates virtually free word-of-mouth marketing. In an environment with increasing consumer distrust of corporate messages, word-of-mouth marketing is potentially a powerful way of increasing consumer confidence. A 2013 international survey found that 84% of people place the most trust in the word-of-mouth recommendations of family and friends.[14] Further, social media facilitates word-of-mouth marketing to ‘go viral’ and spread a message across as many, if not more, consumers than would be reached through traditional broadcast media, often for comparatively little investment.[15]

Public health organisations have recognised that they, too, can make use of social media platforms like Facebook to engage their target market.[16–18] Some of the key benefits of using social media for health communication include: the ability to make health information more available, sharable, and tailored; to provide peer, social, or emotional support; and to influence health policy.[19] Moreover, it appears the public is generally receptive to receiving health messages through social media.[20] However, despite longstanding discussion,[21] there is very little evidence available on the best ways to engage with public health audiences in this space, particularly at the population level.[17, 22] Limited information is available describing non-profit organisations’, including health-related organisations, use of Facebook [18, 23, 24] but, to our knowledge, there has been no examination of what are effective strategies for driving user engagement for these sorts of organisations. Consequently, public health organisations may not be making optimal use of social media platforms like Facebook as part of their overall communication strategy.

There is, however, considerable and expanding evidence demonstrating the effectiveness and importance of population-level health communications.[25] One strategy that has been employed is social marketing, which involves the application of conventional marketing techniques, including advertising and promotion, to achieve a social benefit.[26] These communication techniques have proven particularly effective in tobacco control, where they have been shown to influence attitudes, knowledge, and behaviour change.[27–29] For example, a defining feature of successful tobacco control mass media campaigns has been their emotional appeal, and studies show these types of campaigns have the greatest effect on audiences.[30, 31] Evidence showing benefit for mass media campaigns on other health topics is still developing but is nonetheless generally supportive of the value of population-level health communications.[32, 33]

This study aimed to review the use of Facebook by Australian public health organisations to identify features of their posting activity that are associated with user engagement, which we define as likes, shares, or comments. Specifically, we asked: (1) what communication and conventional marketing techniques are being employed by public health organisations on Facebook? and (2) what techniques are associated with greater user engagement?

## Methods

Two authors (JK and BCF) independently identified a shortlist of Facebook pages relevant to selected public health issues (Table 1). We selected these pages on the basis that the associated public health issues contribute significantly to current levels of morbidity and mortality both in Australia and globally.[34, 35] The shortlist was generated in two phases: an initial search on the social media-monitoring site, Socialbakers [36] and a subsequent search on Google. Socialbakers provides a freely available list of the top 1,000 Facebook pages by ‘likes’ across a range of industries, with the option to filter ‘likes’ by user country. On Facebook, the ‘like’ feature allows users to show support for posted content or pages. We scanned all industries for any public health-related pages with more than 10,000 ‘likes’ by Australian users. To distinguish between liking a post and liking a page, we refer to users who have liked a page as ‘fans’.

**Table 1:** Selected public health issues and related search terms.

Issue	Search terms (Facebook AND…)
Smoking	Smoking, Quit smoking, quitting smoking, tobacco, no tobacco, tobacco free, smoking cessation, give up smoking, lung cancer
Healthy diet	Diet, nutrition, fruit, vegetables, sugar, fat, eat fruit, eat vegetables, fruit and vegetables, healthy eating
Physical activity/sedentariness	Physical activity, exercise, fitness, active travel, physical inactivity, sedentary, sitting, move more, get active
Overweight/obesity	Overweight, obesity, weight loss, weight management, healthy weight, fat, healthy lifestyle
Alcohol	Alcohol, anti-alcohol, drinking, binge drinking, drunk, intoxication, drink driving
Sexual health	Sexual health, HIV, AIDS, contraception, condoms, the pill, HPV, Safe sex, Oral contraceptive pill
Illicit drug use	Drugs, illicit drugs, cannabis, methamphetamine, cocaine, heroin, weed, pot, ecstasy, meth,
Skin cancer	Skin cancer, melanoma, sun protection, ‘slip, slop, slap’, sun safety
Aboriginal Health	Aboriginal health, Indigenous health

To increase the comprehensiveness of our search, we supplemented our Socialbakers search with a Google web search of key terms relating to the selected public health issues. We examined the first three pages of search results for any public health-related Facebook pages, regardless of the number of likes. We excluded pages that were not predominately focussed on a public health issue(s) (e.g. healthcare-related pages) and any commercial pages from the analyses. Once both authors had finalised their shortlist, we compared the lists and resolved discrepancies through discussion or through referral to BF. All searches were conducted in late September 2015.

We further restricted our sample to exclude pages that did not have a primary prevention focus or had less than 10,000 likes by Australian users. The latter criterion was applied so that our analyses were focused on those pages that had already generated comparatively high-levels of interest from Facebook users and for practical reasons; namely to limit the number of pages included in the analyses to an amount that we could manage within available resources. One exception was a page focused on Aboriginal health, which was retained despite not reaching our threshold (this page only had 4,895 Australian fans) because it covers a health priority not explicitly addressed by any of the other eligible pages. A list of the excluded pages and their characteristics is provided in S1 Table.

We recorded descriptive characteristics of each page, including the number of fans, the date of first post, comments/likes/shares per post, the number of users ‘talking about’ a page and calculated the average number of posts made per day and per month, and the median likes, shares, and comments per post. The ‘Talking about’ characteristic represents the number of unique users who have created a story about a page in a 7-day period, and is calculated and updated daily by Facebook. A user ‘creates a story’ when they like a page, post on a page wall, or like, comment, or share a post, among other things. We used this metric to calculate the percentage of people talking about the page as a proportion of the total number of fans to give an indication of the proportion of fans who actively engage with the page beyond just liking it, as done previously.[5]

We developed a coding frame based on that used by Freeman et al. [5], with modifications made during iterative testing to ensure consistency across coders and make the coding frame more relevant to public health communication, as opposed to conventional promotion of commercial goods. Specifically, we added an indicator for the primary communication technique, as defined in Table 2, which captured the messaging style. The coding frame was developed by three of the authors (JK, BCF, and BF) through examining a subset of posts (n = 40) from two of the included pages. Inter-rater reliability was then tested by two authors (JK and BCF) independently coding the same subset of posts (n = 80) from four additional pages. As with page selection, discrepancies were resolved through discussion, with input from a third researcher (BF) where agreement could not be reached. Once inter-rater reliability reached 80%, JK and BCF then individually coded half of eligible pages each. We coded every post generated by the page administrators for a one-year period, from 6 September, 2014 to 31 August, 2015. Multiple marketing elements were possible in a single post, however the predominant communication technique and post-type contained mutually-exclusive categories only.

**Table 2:** Final coding frame with definitions.

Item	Definition
Facebook post type	Whether the post was a photo (or image), text only, game, poll or quiz, app, link, event, or video.
**Communication techniques**^a^	
Informative	Provides information on a health issue, its associated behaviours and/or associated consequences or benefits.
Call-to-action	Encourages users to undertake a specific action (e.g. call a quitline, make an appointment, register for a program or event etc.). A call-to-action was given precedence of instructive or informative messages.
Instructive	Provides instruction on how to do a behaviour.
Positive emotive appeal	Aims to elicit positive emotions like hope and excitement in users. Also includes posts that aim to generate a positive feeling about the brand.
Fear appeal	Aims to elicit fear or other negative emotions in users.
Testimonial	Use of ‘real’ people and/or tells a personal story to encourage behaviour change or to generate emotions about the brand or the health issue. A testimonial was given precedence over emotional appeals.
Humour	Uses any humorous technique (e.g. sarcasm, jokes, memes etc.) to convey a health message
**Marketing elements**	
Branding elements	Any logos, colours, trademarks, or slogans
Celebrities/ sportspeople	People with an entertainment, media, or sports profile who have been linked to the brand. The link could be explicit or implied.
Characters/ mascots	Any characters or mascots developed for the brand
Competitions, prizes, giveaways	Any contest involving a participant entry, including minimal requirements such as liking or commenting on a post.
Person of authority	Any person used for the purpose of lending their personal or positional authority to the brand or health issue (e.g. doctor, academic, scientist, politician).
Sponsorships and partnerships	Any events that the brand supports or other brands with which the brand partners
Vouchers, offers, rebates	Any special deal for brand-related merchandise or events

^a^ The communication technique used in the video or photo was coded first and we only referred to associated text within the post when the technique was not apparent.

We also noted whether the pages allowed user-generated content to be posted and if the page administrators engaged with users either through liking or replying to user comments on their own posts or on user-generated posts. Consistent with Freeman et al. [5], we did not further examine user-generated content because page users are considerably less likely to be exposed to such content as it does not appear in the primary news feed of the page.

We also asked page administrators whether they were willing to share their pages’ Insights data [37] for all page, post, and video data for the same period. Insights data covers a number of metrics not publically available including the total reach of the post (defined as the total number of times the post appeared in a news feed within the first 28 days after posting), the number of clicks on the post, the amount of negative feedback received on a post, and, for videos, the total number of video views.

Excluding the Insights data, all data used in this study are publically available and were collected in accordance with Facebook’s and Socialbakers’ terms and conditions. Insights data were used with the permission of the relevant page administrators.

### Statistical analyses

We generated descriptive statistics for each post type, communication technique, and marketing element. We then investigated associations between the use of each post type, communication technique, and marketing element and the amount of user engagement, operationalized as likes, shares, and comments in this study. To do this, we conducted a series of (separate) negative binomial regressions (the data were over-dispersed) with the count of likes, comments and shares as the outcome variables, and post type, communication technique, and marketing elements as categorical independent variables. The reference category for post-type was photos as this was the most popular category and for communication technique was call-to-action because it represented a concrete action for users to take, as opposed to all of the other categories, which aimed to either inform or evoke emotion. As posts were nested within pages, a variable indicating the page on which the post was published was also included in the models. In addition, we conducted post-estimation contrasts of the effect for each page compared with mean as there was no one page that could sensibly serve as a reference category.

For the subset of pages for which we had Insights data (n = 9) we examined the descriptive characteristics of posts, including the total reach (number of unique users to whom the post was shown) and impressions (total number of times the post appeared in all users’ newsfeeds), as well as the number of post consumers (unique users who clicked anywhere on the post), link clicks (unique users who clicked on a link), video plays (unique users who clicked ‘play’ on a video), and video views (unique users who watched the video for 30 seconds or until the end, whichever came first). Video views includes both users who actively clicked ‘play’ on a video and those who viewed the video as a result of Facebook’s ‘auto-play’ feature, which will automatically start playing a video when a user scrolls through their newsfeed.

In addition, we used the Insights data to identify significant correlates of post-engagement while controlling for users’ exposure to the post; that is by including an exposure or “offset” variable, we can estimate engagement with a post (ie., likes, comments etc) whilst accounting for the different number of people each post is delivered to or times the post appears in a newsfeed.[38] The relationship between post characteristics and engagement therefore becomes a rate per exposure. To do this, we re-ran the models described above using a number of offsets, namely reach and impressions and fan-reach and fan-impressions (reach and impression measures restricted to the fan population only). In addition to likes, comments, and shares, the number of post consumers was used as an outcome variable as another way of operationalizing engagement.

All analyses were conducted using Stata 14.1. Results for all regression are presented as incident rate ratios (IRR) with 95% confidence intervals.

## Results

Our initial search returned 63 eligible pages, which was reduced to a final list of 22 included pages after elimination of those with less than 10,000 fans (Table 3). Two pages, ‘*Be the influence*: *Tackling binge drinking’* and ‘*Shape Up Australia’*, had no posts during the study period, leaving 20 pages for further analysis.

**Table 3:** Characteristics of included Facebook pages.

Page name	Public health issue	Number of Australian fans (total fans)	Date of first post	Average posts per day^a^	Average posts per month^a^	Number of users talking about the page^b^ (as a % of total fans)	Median likes per post (as a % of total fans)	Median shares per post (as a % of total fans)	Median comments per post (as a % of total fans)
beyondblue	Mental health	384,121 (465,839)	11 July 2012	0.5	14.5	17,564 (3.8%)	2089.0 (0.45%)	421.5 (0.09%)	63.5 (0.01%)
R U OK Day	Mental health	282,293 (303,171)	28 Sept 2010	0.6	19.1	4,493 (1.5%)	467.5 (0.15%)	100.5 (0.03%)	13.0 (0.00%)
Be the influence: tackling binge drinking	Alcohol	151,533 (165,655)	14 Feb 2010	0.0	0.0	211 (0.1%)	N/A	N/A	N/A
headspace	Mental health	73,922 (84,221)	26 Aug 2009	0.8	23.0	1,390 (1.7%)	177.0 (0.21%)	32.0 (0.04%)	4.5 (0.01%)
Reachout.com Australia	Mental health	53,475 (60,689)	26 Oct 2009	1.0	31.2	5,402 (8.9%)	374.5 (0.62%)	40.0 (0.07%)	12.0 (0.02%)
Movember Foundation Australia	Men’s health	45,669 (53,937)	25 Jan 2012	0.8	22.6	2,623 (4.9%)	164.0 (0.30%)	19.0 (0.04%)	5.0 (0.01%)
Mums united—Heart Foundation	Heart disease	41,084 (43,069)	19 Aug 2011	0.1	2.3	22 (0.1%)	35.5 (0.08%)	13.5 (0.03%)	1.0 (0.00%)
Cancer Council Australia	Cancer prevention and treatment	39,100 (44,885)	4 Jun 2009	1.4	41.9	795 (1.8%)	33.0 (0.07%)	4.0 (0.01%)	1.0 (0.00%)
Liptember	Mental health	36,322 (37,799)	16 May 2010	0.2	5.1	1,135 (3.0%)	48.0 (0.13%)	2.0 (0.01%)	3.0 (0.01%)
How to drink properly	Alcohol	31,612 (33,125)	20 Feb 2014	0.1	2.3	17 (0.1%)	463.0 (1.40%)	19.5 (0.06%)	78.0 (0.24%)
Cancer Council NSW	Cancer prevention and treatment	30,062 (36,087)	24 May 2010	1.3	40.2	3,557 (9.9%)	111.0 (0.31%)	14.5 (0.04%)	2.0 (0.01%)
SunSmart	Skin cancer prevention	26,017 (28,018)	13 Nov 2011	0.5	14.6	101 (0.4%)	15.0 (0.05%)	2.0 (0.01%)	0.0 (0.00%)
Heart Foundation	Heart disease	25,408 (31,459)	20 Sept 2010	0.8	24.4	3,197 (10.2%)	51.0 (0.16%)	11.0 (0.03%)	1.0 (0.00%)
Quit Victoria	Smoking	20,611 (21,785)	27 Jul 2010	0.5	13.8	99 (0.5%)	32.0 (0.15%)	2.0 (0.01%)	4.0 (0.02%)
Cancer Council Queensland	Cancer prevention and treatment	18,467 (19,076)	18 Feb 2010	1.7	49.8	1,090 (5.7%)	25.5 (0.13%)	1.0 (0.01%)	0.0 (0.00%)
Shape Up Australia	Obesity	16,502 (17,545)	2 Jan 2013	0.0	0.0	21 (0.1%)	N/A	N/A	N/A
Hello Sunday Morning	Alcohol	14,407 (33,029)	1 Jan 2010	0.5	13.8	84 (0.3%)	251.0 (0.76%)	19.0 (0.06%)	12.0 (0.04%)
Make Smoking History WA	Smoking	13,118 (13,663)	19 Aug 2012	0.4	11.8	64 (0.5%)	19.0 (0.09%)	0.0 (0.00%)	1.0 (0.01%)
Ending HIV	Sexual health	12,898 (14,496)	1 Jan 2010	2.0	61.0	354 (2.4%)	13.0 (0.09%)	1.0 (0.01%)	0.0 (0.00%)
Nutrition Australia	Nutrition	12,826 (15,024)	14 Feb 2011	0.4	12.5	737 (4.9%)	36.0 (0.24%)	8.5 (0.06%)	1.5 (0.01%)
Pretty Shady	Skin cancer prevention	11,136 (11,631)	7 Nov 2013	0.1	4.4	7 (0.1%)	78.0 (0.67%)	1.0 (0.01%)	6.0 (0.05%)
Naccho Aboriginal Health Australia	Aboriginal health	4,895 (5,143)	27 Mar 2012	1.3	37.5	1,243 (24.2%)	21.5 (0.42%)	2.0 (0.04%)	0.0 (0.00%)

^a^ Calculated for the period 6 September, 2014 to 31 August 2015

^b^ The number of unique users who have created a story about a page in a 7-day period, calculated and updated daily by Facebook. A user creates a story when they like a page, post on a page wall, and like, comment, or share a post, among other things.

Included pages had a median of 28,040 Australian fans and 33,077 total fans; had been active for on average 4.5 years; and averaged 0.5 posts per day with a range of 0.1 to 2.0. An average of 2.3% of fans had talked about the page in the last seven days. Almost all pages were administered by a non-government organisation and mental health (n = 5) and cancer prevention (n = 5) were the most common public health issues. Over three-quarters of the pages allowed user-generated content (77%) and 86% engaged in conversations with fans. Having a mental health focus attracted the highest number of fans, with four of the top five most-liked pages focusing on this issue. Additionally, the top two pages, ‘*beyondblue’* and *‘R U OK Day’*, were ranked inside the top 1,000 most liked Facebook pages in Australia, according to Socialbakers.

In total, we coded 5,356 posts. Most posts were photos (or images), with the next most common being links (Table 4). Very few posts were apps, games, polls or quizzes, or events. The most common communication technique was a positive emotional appeal, closely followed by testimonial, while the least common was the use of fear appeal. Only half of the posts contained any marketing elements.

**Table 4:** Frequencies of types of post, communication techniques, and use of marketing elements, all pages combined (n = 5356).

	n (%)		n (%)
**Type of post**			
Photos (or images)	3,802 (71.0)	Links	1,286 (24.0)
Videos	181 (3.4)	Text only	80 (1.5)
Other	7 (0.1)		
**Communication technique**			
Positive emotional appeal	1,811 (33.8)	Testimonial	1,366 (25.5)
Call-to-action	750 (14.0)	Informative	674 (12.6)
Humour	425 (7.9)	Instructive	278 (5.2)
Fear appeal	52 (1.0)		
**Marketing elements**^a^			
Branding elements	2,047 (38.7)	Sponsorships or partnerships	576 (10.8)
Celebrities and sportspeople	241 (4.5)	Person of authority	123 (2.3)
Competitions, prizes, or giveaways	107 (2.0)	Characters or mascots	52 (1.0)
Vouchers, offers, or rebates	32 (0.6)	None	2,655 (49.6)

^a^ Marketing elements were not mutually exclusive so total will not add to 100%

On average, over all pages, video posts received on average 25% more likes than photo posts, while links and text posts received 37% and 31% fewer likes respectively (Table 5). Shares displayed a similar pattern, with videos receiving nearly four times as many likes as photo posts on average, while links and text received 30% and 69% fewer shares, respectively. Video and text only posts received more comments on average than photo posts (IRR = 2.03 and 1.59, respectively).

**Table 5:** Associations between post type, communication techniques, and use of marketing elements with engagement metrics, all pages combined.

	Likes		Shares		Comments	
	Incident rate ratio (IRR)	95% conf. interval (CI)	IRR	95% CI	IRR	95% CI
**Post type**						
Photo	Ref		Ref		Ref	
Links	0.63	0.58–0.69	0.70	0.60–0.83	0.85	0.73–1.00
Videos	1.25	1.05–1.48	3.83	2.86–5.12	2.03	1.56–2.63
Text only	0.69	0.55–0.87	0.31	0.21–0.47	1.59	1.11–2.28
**Communication technique**						
Call-to-action	Ref		Ref		Ref	
Fear appeal	1.26	0.93–1.72	1.00	0.60–1.66	1.72	1.06–2.79
Humour	1.08	0.92–1.28	0.30	0.24–0.39	2.01	1.54–2.61
Informative	1.09	0.98–1.22	2.18	1.77–2.70	1.16	0.96–1.39
Instructive	0.93	0.80–1.08	0.88	0.67–1.14	0.77	0.60–0.98
Positive emotional appeal	1.18	1.08–1.30	0.73	0.62–0.86	0.90	0.77–1.04
Testimonial	1.09	0.99–1.20	0.41	0.35–0.48	0.87	0.74–1.01
**Marketing elements**						
Branding elements	1.06	0.99–1.14	0.90	0.80–1.02	0.82	0.73–0.92
Sponsorships and partnerships	0.59	0.54–0.65	0.42	0.35–0.50	0.50	0.43–0.60
Celebrities and sportspeople	1.62	1.41–1.86	2.59	2.01–3.34	1.64	1.30–2.05
Person of Authority	0.80	0.66–0.96	0.50	0.37–0.70	0.71	0.52–0.97
Competitions, prizes, or giveaways	0.71	0.57–0.88	0.43	0.30–0.64	4.05	2.86–5.74
Characters or mascots	0.66	0.49–0.87	0.67	0.40–1.13	2.56	1.60–4.09
Vouchers, offers, or rebates	0.84	0.59–1.21	0.43	0.23–0.81	0.90	0.50–1.59

With regards to communication technique, posts that made use of positive emotional appeal received on average 18% more likes than call-to-action posts but 27% fewer shares. Humorous posts and testimonials also received fewer shares than call-to-action (IRR = 0.30 and 0.73 respectively), while informative posts received more than twice as many shares but with no effect observed for likes or comments. Both fear appeal and humorous posts received more comments on average than call-to-action posts (IRR = 1.72 and 2.01, respectively), while instructive posts received 23% fewer.

Posts with celebrities and sportspeople generally received a greater level of engagement, receiving 62% more likes, two and a half times the number of shares and 64% more comments than posts without celebrities and sportspeople. Most other marketing elements tended to receive either fewer likes, shares, and comments than posts without these elements, or there was no association. The only exceptions to this were for competitions, prizes, and giveaways and characters or mascots, which received significantly more comments on average than posts without these elements (IRR = 1.64, 4.05, and 2.56, respectively).

The frequencies of types of post, communication techniques, and use of marketing elements for posts for which we were able to obtain Insights data (n = 1,563) were similar to the complete sample. Median impressions and reach were greatest for video posts and text only posts, instructive and testimonial posts, and characters or mascots and celebrities and sportspeople (Table 6). Regardless of the post type, communication technique employed, or marketing element used, only 2% to 6% of potential consumers engaged with it in some way.

**Table 6:** Median reach, impressions, post consumers, video plays, and video views of posts with Insights data (n = 1,563).

	Median impressions per post	Median reach per post	Median post consumers per post (as a % of reach)	Median link clicks/video plays per post (as a % of reach)	Median video views per post (as a % of reach)
**Post type**					
Photos	7,173	3,772	111 (3%)	-	-
Links	6,303	3,211	80 (2%)	36 (1%)	-
Videos	42,129	34,608	705 (2%)	553 (2%)	1,863 (5%)
Text only	16,534	8,675	287 (3%)	-	-
**Communication technique**					
Call-to-action	6893	3506	81 (2%)	-	-
Fear appeal	4857	3360	97 (3%)	-	-
Humour	4889	2441	81 (3%)	-	-
Informative	6820	3394	93 (3%)	-	-
Instructive	9094	4674	136 (3%)	-	-
Positive emotional appeal	7453	3910	114 (3%)	-	-
Testimonial	8743	4919	157 (3%)	-	-
**Marketing elements**					
No marketing elements	6438	3317	97 (3%)	-	-
Branding elements	7741	4126	114 (3%)	-	-
Sponsorships and partnerships	10522	5363	168 (3%)	-	-
Celebrities and sportspeople	11869	7067	197 (3%)	-	-
Person of Authority	7008	3281	161 (5%)	-	-
Competitions, prizes, or giveaways	7841	4116	120 (3%)	-	-
Characters or mascots	47534	26448	1590 (6%)	-	-
Vouchers, offers, or rebates	8222	5377	130 (2%)		

When we analysed this subset of posts using the Insights data to account for total reach and impressions and fan-reach and impressions, video posts consistently received fewer likes, shares, and comments per unique user reached and per impression, compared to photo posts (S2–S4 Tables), in contrast to the analysis without the offset. Humorous posts also consistently received fewer likes, shares and comments per user reached and per impression, while positive emotional appeal posts generally received more likes and shares, but not comments, than calls-to-action. The sub-analysis also consistently showed that having a celebrity or sportsperson in a post either made no difference to the number of likes, shares, or comments per user reached or per impression or the association was reversed and they received fewer likes, shares, or comments.

In the analysis where the outcome of interest was post consumers, video posts were significantly more likely to receive any interaction than photo posts when accounting for fan impression and reach, but not when accounting for all impressions and reach (S5 Table). Links and text only posts consistently received fewer post consumers than photo posts, regardless of offset. Testimonial-style posts had a greater number of post consumers per impression and per unique user compared to call-to-action posts, while humour and instructive posts received fewer post consumers per fan impression and unique fan. Branding elements were found to have a mixed relationship with post consumers, receiving fewer per impression and unique user than posts without branding elements but receiving more per fan impression and unique fan. On the other hand, sponsorships and partnerships and persons of authority had fewer post consumers per fan impression and unique fan.

## Discussion

This study has identified some of the characteristics of public health-related Facebook posts that are associated with increased or decreased user engagement. Notably, very few fans will actively engage with any one post, with median likes per post as a percentage of total fans ranging between 0.05% and 1.4%. This reinforces the need for posting content that maximises the chance of high engagement if an organisation is to have any opportunity to make a meaningful impact on public health outcomes on social media. The results presented in this paper provide public health organisations some guidance on how they may improve engagement with social media users.

Our results showed that video posts were the most engaging post type, shared on average four times more often than photo posts. This suggests that fans are more likely to see video posts as novel, interesting, and worthy of sharing with their friends, which is in line with current industry predictions about the value of video for any content provider.[39] However, only 3% of all posts we coded were videos suggesting that public health organisations are trailing behind conventional marketers, with Cisco predicting that video will account for 69% of all consumer internet traffic by 2017 and 80% by 2019.[40]

Conversely, links and text-only posts received fewer likes and shares than photo posts, implying that these posts are generally not seen as engaging, regardless of the content. Lack of engagement with links may be because Facebook users are reluctant to leave Facebook for an external site.[41] Our results suggest that links in particular do not promote engagement, especially when it seems that links are generally reach the fewest number of people of all types of posts and only 1% of these users actually click on the link.

When we accounted for the exposure that people have to a post, the strong effect on engagement of having a video post compared to photo posts was reversed indicating that, per impression, a photo attracted greater engagement and the very high reach of video posts is perhaps what accounts for their popularity. Although Facebook has revealed little about the algorithm which determines the amount of exposure a post receives, [8–10] it is clear that it is complicated and multifactorial, depending not only on the form the post takes but a dynamic combination of factors taking into account each individual’s engagement patterns (like, share or comment) with that particular post.[42] All of this underlines the need for public health organisations to invest significant resources into the management of their Facebook page so that quality content is given the best chance of success. This may include developing a social media (including Facebook) strategy, ensuring resources are available to develop content, and having a dedicated staff member or team to design, implement, and evaluate all social media activity.

Our analysis showed that many traditional marketing elements were associated with lower levels of engagement. In particular, sponsorships and partnerships and use of persons of authority resulted in fewer likes, shares, and comments, compared to posts with no marketing elements at all. On the other hand, the use of celebrities and sportspeople resulted in higher levels of engagement on average, although this relationship was either not significant or reversed when accounting for reach and impressions. Some research from commercial marketing has suggested that celebrities can have significant impact on brand awareness and affinity[43] but that there is also a risk that the celebrity will overshadow the brand.[44] Given this, we recommend further research looking at the role of celebrity in public health marketing on social media, particularly in relation to why fans are more likely to engage with posts that contain celebrities or sportspeople and the effect the use of celebrity has on receptivity to the public health message being conveyed.

Results for the use of different communication techniques were less clear, however. Positive emotional appeal posts, for example, received on average more likes but fewer shares than call-to-action posts, suggesting that these posts prompt only minimal levels of engagement from fans. Humorous posts, however, attracted significantly fewer likes and shares but more comments. This is may be due to the highly subjective nature of humour; that is, what some fans would consider funny could differ wildly from other fans, leading to either no engagement with many fans or negative engagement. We speculate that the reason humorous posts (and also fear appeal posts) received more comments on average is due to their controversial nature. While we did not systematically examine the content of user comments, we did note in coding the posts that many of these types of posts contained negative comments or comments that indicated behaviour in conflict with the intent of the post. For example, a humorous post aimed at discouraging excessive consumption of alcohol included many comments from fans bragging about how they regularly consumed alcohol in excess and would continue to do so.

Emotive posts, particularly positive emotional appeals, were the most common post types, perhaps reflecting research on public health messages disseminated through traditional mass media that shows emotive messages prompt the greatest response from viewers.[30, 45] Research in commercial marketing on Facebook also supports this, with persuasive content, including content aimed at eliciting positive emotions, found to have a positive impact on engagement.[46] The same research also found that informative content had a negative impact except if used in conjunction with persuasive content. However, our analysis found that informative posts provoked more engagement, being shared more than twice as often as call-to-action posts. It may be that posts that include new information about a public health issue prompt a higher level of interest and engagement from fans. Alternatively, it may be because public health organisations are creating emotive content that either fails to generate sufficient emotion in fans to encourage engagement beyond liking a post or is targeting the ‘wrong’ emotions. Another possible explanation could be that Facebook users engaging with public health-related pages do so for different reasons than they would with commercial pages. Understanding the emotions that people feel when exposed to public health-related content and what these emotions prompt them to do is therefore worthy of further research.

Generating a large amount of likes, shares, and/or comments, while an indication of interesting content, should not be seen as the most important outcome of a social media campaign.[41] In theory, engagement with public health pages on Facebook will lead to the achievement of public health aims but this is yet to be proven. There is, however, some evidence from the commercial sphere that engagement with Facebook pages leads to increased sales and profitability,[47, 48] with one study finding that likes are the strongest indicator of long-term sales.[49] Although the generalisability of those findings is limited, together they suggest that simply being seen is not enough and that organisations should only be using Facebook where they are willing and able to invest sufficient resources to engage users. Further research could assist in understanding whether engagement with public health-related pages on Facebook (and social media more broadly) actually leads to the achievement of public health goals.

One issue that we could not explore in this study is the importance of the nature of the page itself. For instance, pages dedicated to, or with a strong focus on, mental health dominated our list of included pages. Furthermore, they made up four of the top five pages in terms of number of fans, suggesting that there is something about mental health that lends itself to the Facebook platform. Other issues, like physical activity and overweight and obesity, were conspicuous by their absence, as were government-run pages. To our knowledge, there is has been no investigation of the suitability and acceptability of particular health issues for Facebook communications. Future studies could sample more pages within each health issue to clarify the effect of health issue on engagement.

Limitations of this paper include using a previously untested coding framework for identifying the communication techniques used. We did, however, employ a rigorous development and testing regime to increase the chances of high inter-rater reliability between the two coders. Another limitation was that for practical reasons our analysis only considered pages with 10,000 or more fans, which was an arbitrary cut point. It is possible that pages with fewer fans operate in markedly different ways than the pages we considered here, which may contribute to them having fewer fans or there may other factors independent of content that account for the size of the fan base. We also could only obtain Insights data on less than half the pages eligible for our study, limiting our analysis of these fine-grained measures. Finally, it is worth noting that our findings may not be generalizable to other social media platforms. This is due to users having differing motivations and expectations for using particular platforms. [11, 50] Additional research with other platforms is necessary to understand what works best and to explore whether there are commonalities across platforms.

## Conclusions

Our results are a necessary first step in filling the knowledge gap on the effective use of Facebook by public health organisations. By critically examining the characteristics of Facebook posts created by Australian-based public health organisations, we have identified post types and marketing techniques that attract greater or lesser user engagement. Further research will be essential, particularly in relation to whether certain health issues (e.g. mental health) are better suited to Facebook. Our study has shown that in order to increase the chances of achieving public health goals, content providers must encourage engagement and adapt to the Facebook algorithm in order to maximise message exposure, while also ensuring that the content is of high quality. Our study will assist public health organisations to use this powerful platform more efficiently and effectively.

## Supporting Information

S1 Data (XLSX)

S1 Table Key characteristics of excluded pages. (DOCX)

S2 Table Associations between post type, social advertising techniques, and use of marketing elements with likes, with offsets for impressions and reach. (DOCX)

S3 Table Associations between post type, social advertising techniques, and use of marketing elements with shares per impression and unique user. (DOCX)

S4 Table Associations between post type, social advertising techniques, and use of marketing elements with comments per impression and unique user. (DOCX)

S5 Table Associations between post type, social advertising techniques, and use of marketing elements with post consumers per impression and unique user. (DOCX)
