# Supplementary material for: Please Like Me: Facebook and Public Health Communication
Source: PLoS One. 2016 Sep 15;11(9):e0162765. doi: 10.1371/journal.pone.0162765 (PMC5025158; doi:10.1371/journal.pone.0162765)
Supplement: S1 Table — (DOCX) [file pone.0162765.s002.docx]

Table S1 Key characteristics of excluded pages

| **Page name** | **Public health issue** | **Number of Australian fans (total fans)** | **Reason for exclusion** |
| --- | --- | --- | --- |
| McGrath Foundation | Cancer treatment | 281,529 (304, 404) | Not primary prevention |
| Bowel Cancer Australia | Cancer screening | 54,075 (55,129) | Not primary prevention |
| Close the Gap | Aboriginal health | 10,583 (11,004) | Not primary prevention |
| 8700.com.au | Nutrition | 9,678 (10,623) | Less than 10,000 Australian fans |
| Live_lighter | Overweight/obesity | 8,782 (9,191) | Less than 10,000 Australian fans |
| Australian Drug Foundation | Illicit drugs | 6,573 (6,890) | Less than 10,000 Australian fans |
| Odd Socks Day | Mental health | 5,535 (5,815) | Less than 10,000 Australian fans |
| Melanoma Institute Australia | Skin cancer | 4,953 (5,809) | Less than 10,000 Australian fans |
| TeamUp Victoria | Physical activity | 4,948 (5,114) | Less than 10,000 Australian fans |
| Make Healthy Normal | Overweight/obesity | 4,898 (4,984) | Less than 10,000 Australian fans |
| Yeah! Youth Empowerment Against HIV/AIDS | Sexual health | 4,319 (5,726) | Less than 10,000 Australian fans |
| Ending HIV Queensland | Sexual health | N/A (3,879) | Less than 10,000 Australian fans |
| Australian Cervical Cancer Foundation (ACCF) | Cancer screening | 3,080 (3,648) | Less than 10,000 Australian fans |
| Walk to Work Day | Physical activity | 2,477 (2,801) | Less than 10,000 Australian fans |
| Heart Foundation Walking | Physical activity | 2,126 (2,346) | Less than 10,000 Australian fans |
| Healthy Together Geelong | Overweight/obesity | 1,891 (1,932) | Less than 10,000 Australian fans |
| Healthy Together Mildura | Overweight/obesity | 1,710 (1,762) | Less than 10,000 Australian fans |
| Drug Aware | Illicit drugs | 1,665 (1,942) | Less than 10,000 Australian fans |
| Active Melbourne | Physical activity | 1,630 (1,813) | Less than 10,000 Australian fans |
| Ending HIV Victoria | Sexual health | 1,572 (1,650) | Less than 10,000 Australian fans |
| Australian Federation of AIDS Organisations (AFAO) | Sexual health | 1,518 (9,448) | Less than 10,000 Australian fans |
| Alcohol. Think again | Alcohol | N/A (1,505) | Less than 10,000 Australian fans |
| Eat Well Tasmania | Nutrition | 1,397 (1,441) | Less than 10,000 Australian fans |
| Australian Council on Smoking and Health | Smoking | 1,213 (1,423) | Less than 10,000 Australian fans |
| MOVE it Bundaberg | Physical activity | 1,268 (1,291) | Less than 10,000 Australian fans |
| Obesity Policy Coalition | Overweight/obesity | 1,196 (1,608) | Less than 10,000 Australian fans |
| Quitline South Australia | Smoking | 1,169 (1,098) | Less than 10,000 Australian fans |
| Your move | Physical activity | N/A (965) | Less than 10,000 Australian fans |
| Healthy Together Wodonga | Overweight/obesity | N/A (928) | Less than 10,000 Australian fans |
| Aboriginal Quitline | Smoking | N/A (744) | Less than 10,000 Australian fans |
| Drinkwise Australia | Alcohol | N/A (666) | Less than 10,000 Australian fans |
| Get moving Tasmania | Physical activity | N/A (621) | Less than 10,000 Australian fans |
| Healthy Together Greater Dandenong | Overweight/obesity | N/A (517) | Less than 10,000 Australian fans |
| AVN | Immunisation | N/A (402) | Less than 10,000 Australian fans |
| Physical Activity Foundation | Physical activity | N/A (388) | Less than 10,000 Australian fans |
| Healthy together Cardinia Shire | Overweight/obesity | N/A (325) | Less than 10,000 Australian fans |
| My health balance & get on track challenge | Overweight/obesity | N/A (322) | Less than 10,000 Australian fans |
| FARE Australia | Alcohol | N/A (312) | Less than 10,000 Australian fans |
| Obesity Australia Ltd | Overweight/obesity | N/A (310) | Less than 10,000 Australian fans |
| CO-OPS Collaboration | Overweight/obesity | N/A (254) | Less than 10,000 Australian fans |

NB: The number of Australian fans was not available for pages some pages
