# Supplementary material for: Please Like Me: Facebook and Public Health Communication
Source: PLoS One. 2016 Sep 15;11(9):e0162765. doi: 10.1371/journal.pone.0162765 (PMC5025158; doi:10.1371/journal.pone.0162765)
Supplement: S2 Table — (DOCX) [file pone.0162765.s003.docx]

Table S2 Associations between post type, communication techniques, and use of marketing elements with likes, with offsets for impressions and reach (n=1,563 posts)

| **Offset** | **No offset**  **IRR (95% CI)** | **Per impression**  **IRR (95% CI)** | **Per unique user**  **IRR (95% CI)** | **Per fan impression**  **IRR (95% CI)** | **Per unique fan**  **IRR (95% CI)** |
| --- | --- | --- | --- | --- | --- |
| **Post type** |  |  |  |  |  |
| Photo | Ref |  |  |  |  |
| Links | 0.74 (0.65-0.86) | 0.56 (0.50-0.63) | 0.56 (0.50-0.63) | 0.25 (0.20-0.30) | 0.24 (0.19-0.30) |
| Videos | 1.68 (1.29-2.19) | 0.30 (0.23-0.36) | 0.29 (0.24-0.35) | 0.33 (0.23-0.48) | 0.29 (0.20-0.42) |
| Text only | 0.88 (0.62-1.25) | 0.50 (0.37-0.66) | 0.53 (0.40-0.70) | 0.17 (0.11-0.28) | 0.18 (0.11-0.30) |
| **Communication technique** |  |  |  |  |  |
| Call-to-action | Ref |  |  |  |  |
| Fear appeal | 1.39 (1.01-1.91) | 0.95 (0.74-1.23) | 0.89 (0.69-1.15) | 0.77 (0.49-1.21) | 0.57 (0.36-0.91) |
| Humour | 0.82 (0.62-1.09) | 0.96 (0.77-1.21) | 0.97 (0.78-1.22) | 0.42 (0.28-0.62) | 0.30 (0.20-0.45) |
| Informative | 1.24 (1.04-1.47) | 0.89 (0.77-1.02) | 0.91 (0.78-1.05) | 0.63 (0.48-0.81) | 0.48 (0.37-0.64) |
| Instructive | 0.84 (0.68-1.03) | 0.82 (0.69-0.97) | 0.81 (0.68-0.96) | 0.45 (0.34-0.61) | 0.35 (0.26-0.48) |
| Positive emotional appeal | 1.54 (1.31-1.80) | 1.50 (1.32-1.70) | 1.51 (1.33-1.73) | 2.11 (1.68-2.64) | 1.57 (1.24-2.00) |
| Testimonial | 1.28 (1.09-1.51) | 1.06 (0.93-1.21) | 1.02 (0.89-1.17) | 0.84 (0.66-1.07) | 0.61 (0.48-0.78) |
| **Marketing elements** |  |  |  |  |  |
| No marketing elements | Ref |  |  |  |  |
| Branding elements | 1.21 (1.08-1.35) | 1.16 (1.06-1.28) | 1.13 (1.03-1.25) | 2.14 (1.81-2.52) | 2.55 (2.15-3.03) |
| Sponsorships and partnerships | 0.77 (0.66-0.90) | 0.82 (0.73-0.93) | 0.84 (0.74-0.95) | 0.45 (0.36-0.57) | 0.44 (0.35-0.55) |
| Celebrities and sportspeople | 0.89 (0.69-1.13) | 0.83 (0.67-1.01) | 0.79 (0.64-0.97) | 1.09 (0.75-1.59) | 1.00 (0.68-1.47) |
| Person of Authority | 0.49 (0.32-0.75) | 0.75 (0.53-1.05) | 0.75 (0.64-0.97) | 0.36 (0.20-0.65) | 0.37 (0.20-0.67) |
| Competitions, prizes, or giveaways | 0.90 (0.64-1.29) | 0.42 (0.31-0.55) | 0.40 (0.30-0.53) | 0.42 (0.26-0.68) | 0.37 (0.23-0.61) |
| Characters or mascots | 0.57 (0.40-0.81) | 0.80 (0.60-1.07) | 0.80 (0.60-1.07) | 0.46 (0.28-0.74) | 0.37 (0.22-0.60) |
| Vouchers, offers, or rebates | 0.70 (0.38-1.27) | 0.64 (0.39-1.05) | 0.60 (0.37-0.99) | 0.49 (0.21-1.13) | 0.51 (0.21-1.24) |
