# Supplementary material for: Please Like Me: Facebook and Public Health Communication
Source: PLoS One. 2016 Sep 15;11(9):e0162765. doi: 10.1371/journal.pone.0162765 (PMC5025158; doi:10.1371/journal.pone.0162765)
Supplement: S3 Table — (DOCX) [file pone.0162765.s004.docx]

Table S3 Associations between post type, communication techniques, and use of marketing elements with shares per impression and unique user (n=1,563 posts)

| **Offset** | **No offset**  **IRR (95% CI)** | **Per impression**  **IRR (95% CI)** | **Per unique user**  **IRR (95% CI)** | **Per fan impression**  **IRR (95% CI)** | **Per unique fan**  **IRR (95% CI)** |
| --- | --- | --- | --- | --- | --- |
| **Post type** |  |  |  |  |  |
| Photo | Ref |  |  |  |  |
| Links | 0.74 (0.57-0.96) | 0.69 (0.57-0.84) | 0.69 (0.57-0.84) | 0.41 (0.31-0.54) | 0.37 (0.27-0.49) |
| Videos | 3.02 (1.88-4.87) | 0.36 (0.26-0.51) | 0.36 (0.26-0.50) | 0.64 (0.39-1.06) | 0.55 (0.33-0.91) |
| Text only | 0.34 (0.19-0.63) | 0.33 (0.21-0.53) | 0.35 (0.22-0.57) | 0.13 (0.06-0.24) | 0.13 (0.06-0.25) |
| **Communication technique** |  |  |  |  |  |
| Call-to-action | Ref |  |  |  |  |
| Fear appeal | 1.31 (0.76-2.24) | 1.83 (1.19-2.82) | 1.71 (1.11-2.65) | 1.06 (0.60-1.87) | 0.85 (0.47-1.54) |
| Humour | 0.37 (0.22-0.62) | 0.63 (0.43-0.93) | 0.61 (0.41-0.90) | 0.25 (0.14-0.42) | 0.19 (0.11-0.32) |
| Informative | 1.96 (1.41-2.72) | 1.35 (1.07-1.71) | 1.37 (1.08-1.75) | 1.03 (0.73-1.45) | 0.83 (0.58-1.20) |
| Instructive | 0.87 (0.60-1.25) | 1.02 (0.78-1.35) | 1.03 (0.78-1.36) | 0.53 (0.36-0.78) | 0.41 (0.27-0.63) |
| Positive emotional appeal | 1.03 (0.76-1.39) | 1.24 (1.00-1.54) | 1.23 (0.99-1.53) | 1.39 (1.01-1.92) | 1.11 (0.79-1.56) |
| Testimonial | 0.58 (0.43-0.78) | 0.66 (0.52-0.82) | 0.61 (0.49-0.77) | 0.43 (0.31-0.59) | 0.34 (0.24-0.48) |
| **Marketing elements** |  |  |  |  |  |
| No marketing elements | Ref |  |  |  |  |
| Branding elements | 1.05 (0.85-1.30) | 1.07 (0.92-1.25) | 1.03 (0.88-1.21) | 2.47 (1.96-3.12) | 2.91 (2.27-3.72) |
| Sponsorships and partnerships | 0.77 (0.56-1.06) | 0.59 (0.48-0.74) | 0.60 (0.48-0.75) | 0.47 (0.34-0.65) | 0.46 (0.33-0.65) |
| Celebrities and sportspeople | 0.89 (0.57-1.39) | 0.71 (0.51-1.00) | 0.68 (0.48-0.96) | 1.23 (0.69-2.21) | 1.10 (0.61-2.00) |
| Person of Authority | 0.34 (0.16-0.71) | 0.62 (0.36-1.08) | 0.63 (0.36-1.10) | 0.39 (0.18-0.85) | 0.40 (0.18-0.90) |
| Competitions, prizes, or giveaways | 0.52 (0.27-1.00) | 0.43 (0.26-0.71) | 0.41 (0.25-0.69) | 0.30 (0.15-0.60) | 0.24 (0.12-0.49) |
| Characters or mascots | 0.58 (0.31-1.08) | 1.14 (0.70-1.86) | 1.13 (0.69-1.86) | 0.61 (0.32-1.14) | 0.48 (0.25-0.92) |
| Vouchers, offers, or rebates | 0.39 (0.13-1.07) | 0.67 (0.30-1.50) | 0.68 (0.30-1.53) | 0.41 (0.13-1.25) | 0.37 (0.12-1.16) |
