# Supplementary material for: Please Like Me: Facebook and Public Health Communication
Source: PLoS One. 2016 Sep 15;11(9):e0162765. doi: 10.1371/journal.pone.0162765 (PMC5025158; doi:10.1371/journal.pone.0162765)
Supplement: S4 Table — (DOCX) [file pone.0162765.s005.docx]

Table S4 Associations between post type, communication techniques, and use of marketing elements with comments per impression and unique user (n=1,563 posts)

| **Offset** | **No offset**  **IRR (95% CI)** | **Per impression**  **IRR (95% CI)** | **Per unique user**  **IRR (95% CI)** | **Per fan impression**  **IRR (95% CI)** | **Per unique fan**  **IRR (95% CI)** |
| --- | --- | --- | --- | --- | --- |
| **Post type** |  |  |  |  |  |
| Photo | Ref |  |  |  |  |
| Links | 1.03 (0.82-1.30) | 0.89 (0.73-1.08) | 0.89 (0.72-1.09) | 0.79 (0.61-1.01) | 0.80 (0.62-1.02) |
| Videos | 2.09 (1.41-3.11) | 0.45 (0.32-0.62) | 0.43 (0.31-0.60) | 0.86 (0.58-1.27) | 0.83 (0.56-1.22) |
| Text only | 2.21 (1.30-3.73) | 1.18 (0.76-1.83) | 1.29 (0.82-2.03) | 0.72 (0.43-1.22) | 0.77 (0.45-1.31) |
| **Communication technique** |  |  |  |  |  |
| Call-to-action | Ref |  |  |  |  |
| Fear appeal | 2.11 (1.31-3.41) | 1.41 (0.95-2.10) | 1.33 (0.88-2.00) | 1.45 (0.90-2.34) | 1.44 (0.89-2.34) |
| Humour | 0.65 (0.42-1.01) | 0.86 (0.58-1.28) | 0.85 (0.57-1.27) | 0.44 (0.28-0.71) | 0.43 (0.27-0.70) |
| Informative | 1.59 (1.21-2.08) | 1.18 (0.93-1.49) | 1.20 (0.94-1.53) | 0.88 (0.66-1.16) | 0.88 (0.66-1.17) |
| Instructive | 0.86 (0.62-1.18) | 0.73 (0.55-0.96) | 0.72 (0.54-0.96) | 0.52 (0.37-0.72) | 0.51 (0.37-0.83) |
| Positive emotional appeal | 1.13 (0.88-1.45) | 1.21 (0.97-1.50) | 1.25 (1.00-1.56) | 1.04 (0.81-1.35) | 1.07 (0.82-1.39) |
| Testimonial | 1.28 (0.99-1.66) | 1.04 (0.83-1.30) | 0.99 (0.79-1.25) | 0.80 (0.61-1.05) | 0.75 (0.57-0.99) |
| **Marketing elements** |  |  |  |  |  |
| No marketing elements | Ref |  |  |  |  |
| Branding elements | 1.01 (0.84-1.21) | 1.04 (0.89-1.22) | 1.03 (0.88-1.22) | 1.39 (1.15-1.67) | 1.47 (1.22-1.79) |
| Sponsorships and partnerships | 0.76 (0.60-0.98) | 0.74 (0.60-0.92) | 0.76 (0.61-0.94) | 0.50 (0.39-0.64) | 0.52 (0.40-0.67) |
| Celebrities and sportspeople | 0.69 (0.47-1.01) | 0.73 (0.52-1.03) | 0.69 (0.48-0.98) | 0.58 (0.39-0.86) | 0.55 (0.37-0.83) |
| Person of Authority | 0.66 (0.35-1.27) | 0.99 (0.55-1.77) | 0.98 (0.54-1.78) | 0.58 (0.29-1.19) | 0.58 (0.28-1.18) |
| Competitions, prizes, or giveaways | 5.59 (3.21-9.74) | 1.72 (1.06-2.80) | 1.42 (0.85-2.35) | 3.41 (1.96-5.94) | 3.08 (1.75-5.41) |
| Characters or mascots | 3.21 (1.91-5.38) | 1.51 (0.98-2.33) | 1.50 (0.96-2.34) | 2.81 (1.68-4.67) | 2.86 (1.70-4.82) |
| Vouchers, offers, or rebates | 0.61 (0.24-1.56) | 0.70 (0.32-1.55) | 0.67 (0.30-1.49) | 0.52 (0.20-1.32) | 0.53 (0.21-1.37) |
