# Supplementary material for: Please Like Me: Facebook and Public Health Communication
Source: PLoS One. 2016 Sep 15;11(9):e0162765. doi: 10.1371/journal.pone.0162765 (PMC5025158; doi:10.1371/journal.pone.0162765)
Supplement: S5 Table — (DOCX) [file pone.0162765.s006.docx]

Table S5 Associations between post type, communication techniques, and use of marketing elements with post consumers per impression and unique user (n=1,563 posts)

| **Offset** | **No offset**  **IRR (95% CI)** | **Per impression**  **IRR (95% CI)** | **Per unique user**  **IRR (95% CI)** | **Per fan impression**  **IRR (95% CI)** | **Per unique fan**  **IRR (95% CI)** |
| --- | --- | --- | --- | --- | --- |
| **Post type** |  |  |  |  |  |
| Photo | Ref |  |  |  |  |
| Links | 0.69 (0.58-0.82) | 0.72 (0.66-0.78) | 0.70 (0.64-0.76) | 0.43 (0.36-0.51) | 0.38 (0.32-0.46) |
| Videos | 7.03 (4.95-9.99) | 0.99 (0.86-1.14) | 0.98 (0.85-1.14) | 2.36 (1.73-3.21) | 2.07 (1.50-2.86) |
| Text only | 1.19 (0.77-1.83) | 0.79 (0.64-0.97) | 0.80 (0.65-0.99) | 0.37 (0.25-0.55) | 0.36 (0.24-0.55) |
| **Communication technique** |  |  |  |  |  |
| Call-to-action | Ref |  |  |  |  |
| Fear appeal | 1.31 (0.89-1.93) | 1.23 (1.02-1.47) | 1.16 (0.96-1.39) | 0.88 (0.62-1.26) | 0.72 (0.49-1.06) |
| Humour | 0.73 (0.51-1.04) | 1.05 (0.88-1.24) | 0.99 (0.83-1.18) | 0.51 (0.37-0.71) | 0.38 (0.27-0.54) |
| Informative | 1.57 (1.29-1.96) | 1.12 (1.01-1.24) | 1.11 (1.00-1.24) | 0.94 (0.76-1.16) | 0.76 (0.61-0.95) |
| Instructive | 1.08 (0.84-1.40) | 1.07 (0.95-1.21) | 1.04 (0.91-1.17) | 0.59 (0.47-0.75) | 0.46 (0.35-0.59) |
| Positive emotional appeal | 1.15 (0.94-1.40) | 1.10 (1.00-1.21) | 1.08 (0.99-1.19) | 1.38 (1.15-1.66) | 1.10 (0.90-1.34) |
| Testimonial | 1.97 (1.60-2.43) | 1.38 (1.25-1.52) | 1.30 (1.17-1.43) | 1.19 (0.98-1.45) | 0.91 (0.74-1.12) |
| **Marketing elements** |  |  |  |  |  |
| No marketing elements | Ref |  |  |  |  |
| Branding elements | 0.80 (0.69-0.93) | 0.85 (0.79-0.91) | 0.80 (0.75-0.86) | 1.39 (1.20-1.60) | 1.50 (1.29-1.74) |
| Sponsorships and partnerships | 1.23 (1.01-1.50) | 0.97 (0.88-1.06) | 0.97 (0.89-1.06) | 0.78 (0.65-0.93) | 0.74 (0.61-0.90) |
| Celebrities and sportspeople | 0.93 (0.68-1.26) | 1.02 (0.88-1.17) | 0.97 (0.83-1.12) | 0.87 (0.64-1.17) | 0.83 (0.61-1.13) |
| Person of Authority | 0.62 (0.37-1.03) | 1.14 (0.89-1.47) | 1.15 (0.89-1.49) | 0.51 (0.32-0.83) | 0.52 (0.32-0.86) |
| Competitions, prizes, or giveaways | 1.13 (0.73-1.75) | 0.86 (0.70-1.06) | 0.82 (0.66-1.01) | 0.87 (0.59-1.30) | 0.72 (0.48-1.10) |
| Characters or mascots | 1.14 (0.73-1.77) | 1.40 (1.13-1.73) | 1.42 (1.14-1.77) | 0.73 (0.49-1.07) | 0.64 (0.43-0.96) |
| Vouchers, offers, or rebates | 0.54 (0.26-1.14) | 0.86 (0.61-1.26) | 0.90 (0.62-1.30) | 0.52 (0.26-1.04) | 0.48 (0.23-0.98) |
